# An Effective QoS-Aware Hybrid Optimization Approach for Workflow Scheduling in Cloud Computing

**DOI:** 10.3390/s25154705

**Published:** 2025-07-30

**Authors:** Min Cui, Yipeng Wang

**Affiliations:** College of Computer Science and Technology, Nanjing University of Aeronautics and Astronautics, Nanjing 211106, China; wyp20021006@nuaa.edu.cn

**Keywords:** workflow scheduling, multi-objective optimization, HEFT, WOA, Lévy flight

## Abstract

Workflow scheduling in cloud computing is attracting increasing attention. Cloud computing can assign tasks to available virtual machine resources in cloud data centers according to scheduling strategies, providing a powerful computing platform for the execution of workflow tasks. However, developing effective workflow scheduling algorithms to find optimal or near-optimal task-to-VM allocation solutions that meet users’ specific QoS requirements still remains an open area of research. In this paper, we propose a hybrid QoS-aware workflow scheduling algorithm named HLWOA to address the problem of simultaneously minimizing the completion time and execution cost of workflow scheduling in cloud computing. First, the workflow scheduling problem in cloud computing is modeled as a multi-objective optimization problem. Then, based on the heterogeneous earliest finish time (HEFT) heuristic optimization algorithm, tasks are reverse topologically sorted and assigned to virtual machines with the earliest finish time to construct an initial workflow task scheduling sequence. Furthermore, an improved Whale Optimization Algorithm (WOA) based on Lévy flight is proposed. The output solution of HEFT is used as one of the initial population solutions in WOA to accelerate the convergence speed of the algorithm. Subsequently, a Lévy flight search strategy is introduced in the iterative optimization phase to avoid the algorithm falling into local optimal solutions. The proposed HLWOA is evaluated on the WorkflowSim platform using real-world scientific workflows (Cybershake and Montage) with different task scales (100 and 1000). Experimental results demonstrate that HLWOA outperforms HEFT, HEPGA, and standard WOA in both makespan and cost, with normalized fitness values consistently ranking first.

## 1. Introduction

Workflow technology [1] plays a crucial role in modern computing, with applications spanning a wide range of important fields from business operations to scientific research. In business environments, workflows are extensively used to optimize enterprise process management. In the scientific domain, especially in data-intensive disciplines such as astronomical observation, weather forecasting, medical diagnosis, and bioinformatics analysis, workflows are an indispensable technical means. These scientific workflows are often large-scale and structurally complex, potentially consisting of thousands or even millions of independent and interdependent computational tasks. As a result, they place extremely high demands on the underlying computing infrastructure, requiring powerful computing capabilities, efficient communication mechanisms, and vast storage space for support.

The emergence of cloud computing has provided an ideal solution for workflow execution. Cloud computing can offer three distinct types of cloud service patterns on demand, namely Infrastructure as a Service (IaaS), Platform as a Service (PaaS), and Software as a Service (SaaS) [2]. Users can dynamically configure cloud service resources on the cloud platform through virtualization technology, including processing power, network bandwidth, and storage space, thereby flexibly meeting the execution requirements of different types of workflows. However, in the complex and heterogeneous cloud computing environment, how to efficiently allocate workflow tasks to available virtual resources and determine the execution order of tasks in a given workflow is a highly challenging problem. This is the workflow scheduling problem that has attracted widespread attention from both academia and industry.

Workflow scheduling in cloud environments confronts multi-dimensional challenges. From the resource perspective, infrastructure heterogeneity causes task execution times to vary dramatically across different virtual machine (VM) types, while fluctuations in network bandwidth make it hard to predict inter-task data transfer durations. Regarding Quality of Service (QoS) requirements, users typically seek to optimize several objectives simultaneously—most notably minimizing makespan and lowering monetary cost—yet these goals are inherently conflicting: high-performance VMs accelerate execution but increase expense, whereas low-cost VMs prolong completion time. Moreover, the dynamic, shared nature of clouds further complicates scheduling, actual resource performance can fluctuate due to multi-tenant contention, causing static, estimate-based scheduling decisions to deviate from expected QoS targets at runtime. These challenges make it difficult to directly apply traditional scheduling methods to workflow scheduling in cloud environments.

The workflow scheduling problem is classified as an NP-complete problem and has been extensively studied in other computing paradigms, such as grid computing and cluster computing [3,4]. For example, for a workflow containing N tasks, if there are M available virtual machines, the number of possible task allocation schemes will reach $M^{N}$ kinds. Moreover, in practical application scenarios, when users execute workflows on cloud platforms, they not only consider the completion of the workflow but also pay attention to some QoS requirement indicators (such as the workflow completion time and the cost of executing tasks). Therefore, designing an effective workflow scheduling algorithm that meets users’ specific QoS requirements to find the optimal mapping of workflow tasks to virtual machine resources is a key research direction for executing workflow task scheduling in the field of cloud computing.

The heterogeneous earliest finish time (HEFT) algorithm [5] is a classic list scheduling algorithm and an efficient heuristic algorithm, mainly used for workflow scheduling represented by Directed Acyclic Graphs (DAGs). The core idea of this algorithm is to first calculate the priority of tasks to determine the scheduling order, and then assign tasks to the VMs that can complete them at the earliest time according to the priority order. Its goal is to minimize the overall completion time of the workflow, without focusing on the optimization objective of task execution cost. The Whale Optimization Algorithm (WOA) is a meta-heuristic optimization algorithm proposed by Mirjalili et al. [6] in 2016, which employs the hunting behavior of humpback whales to find near-optimal solutions. The algorithm begins with the initialization of a group of whale individuals, where each whale represents a solution, and the mass of each whale is calculated based on the fitness function used in the algorithm. Individuals with higher fitness function values represent higher quality solutions, thereby guiding the search direction of the whale population. Through continuous iterative search and individual position updates, all individuals eventually converge to an optimal position. WOA is capable of obtaining the global optimum faster than other meta-heuristic algorithms, thus possessing a faster convergence rate. Mangalampalli S et al. [7] has demonstrated that WOA outperforms other meta-heuristic algorithms such as Ant Colony Optimization (ACO), a Genetic Algorithm (GA), and Particle Swarm Optimization (PSO) in handling multi-objective optimization problems in task scheduling.

In the workflow scheduling process, multi-objective optimization problems essentially involve establishing a mathematical model for the scheduling decision algorithm, quantifying users’ QoS requirements, and further optimizing QoS parameters (such as minimizing makespan and cost). The scheduling goal is to seek the optimal or near-optimal mapping of tasks to virtual machines. Therefore, optimizing scheduling decisions in a complex solution space to find high-quality solution, thereby enhancing workflow scheduling performance and meeting users’ multiple QoS requirements, has always been an open exploration problem in the field of workflow scheduling.

Although HEFT and WOA have been widely adopted in the field of workflow scheduling, each suffers from distinct limitations when used in isolation: (a) HEFT optimizes completion time alone and neglects cost, so its single-objective nature cannot meet the diverse QoS requirements in cloud environments; (b) the fully random initialization of standard WOA is prone to local optima, and its convergence speed is highly sensitive to the quality of the initial solution. In this paper, we propose a new hybrid scheduling optimization algorithm, abbreviated as HLWOA, which combines the strengths of HEFT, WOA, and Lévy flight to address the multi-objective optimization problem of minimizing completion time and cost in workflow scheduling within a cloud computing environment. First, we employ the HEFT algorithm to sort tasks based on their upward rank values and allocate virtual machine resources according to the earliest completion time, generating a mapping for a scheduling sequence. Subsequently, in the initialization phase of the WOA algorithm, the solution generated by HEFT is introduced as one of the initial solutions in the population (with other initial solutions generated randomly), thereby enhancing the quality of the initial solutions to accelerate the convergence speed of the WOA algorithm. During the iterative optimization phase of the WOA algorithm, the Lévy flight strategy is innovatively incorporated to enable the WOA algorithm to avoid local optima as much as possible and identify the optimal mapping of tasks to virtual machines. Finally, we conduct experiments using real-world scientific workflows on the cloud computing simulation platform of WorkflowSim. The results demonstrate that HLWOA outperforms the HEFT, HEPGA, and WOA algorithms in terms of scheduling performance, with significant advantages in both makespan and cost.

In summary, our contributions can be concisely stated as follows:

(1) A novel hybrid architecture combining HEFT’s efficiency with WOA’s global search capability.
(2) An improved WOA incorporating Lévy flight to avoid local optima.
(3) An efficient fitness function balancing makespan and cost to meet the different QoS preferences.
(4) Validation of better performance of HLWOA through simulation and comparison with other scheduling algorithms.

The rest of this article is organized as follows. Section 2 briefly discusses the related works. The related models and problem formulation in this work are presented in Section 3. An effective workflow scheduling algorithm called HLWOA is introduced in Section 4. Section 5 shows the results of the simulation experiments and Section 6 concludes this article.

## 2. Related Works

This section provides a comprehensive review of the scheduling methods involved in the field of workflow scheduling, including heuristic-based algorithms, meta-heuristic-based algorithms, hybrid scheduling methods, and deep learning methods.

HEFT [8] is a classic list-based heuristic algorithm for workflow scheduling, originally developed for task scheduling in heterogeneous multiprocessor systems. It is widely recognized that the HEFT algorithm outperforms other heuristic algorithms such as Min-min and Max-min [9,10] in terms of scheduling performance. However, HEFT focuses solely on optimizing makespan and is essentially a single-objective optimization algorithm. Ahmed S et al. [11] proposed a modified heterogeneous earliest finish time algorithm, called EM-HEFT, which optimized three objectives, namely completion time, resource utilization, and load balancing. Medara R et al. [12] designed an energy-efficient heuristic algorithm (ECWS) based on the HEFT for workflow scheduling, which aimed to decrease energy utilization, execution cost, and maximizing utilization of the resources. Nooriantalouki R et al. [13] proposed a new list scheduling heuristic algorithm that employed the OCTd and OCTu task prioritization strategies and applied task duplication techniques based on the HEFT algorithm, thereby significantly reducing the makespan. In order to minimize the total cost and total idle rate for deadline-constrained workflow scheduling in cloud, Sun Z et al. [14] developed a hybrid heuristic algorithm called enhanced task-type first algorithm (ET2FA). Heuristic algorithms provide a good solution in workflow scheduling optimization, but most heuristic scheduling algorithms are based on a finite solution space for search, and the resulting solution is likely not the better solution.

The meta-heuristic algorithms introduce new solution ideas for the scheduling optimization of workflow tasks, which are used to find approximate optimal solutions for complex optimization problems within a reasonable time [15,16]. Xia X et al. [17] applied a multi-objective Genetic Algorithm (MOGA) to optimize workflow scheduling problems. An initialization scheduling sequence scheme is proposed to realize a proper trade-off between the makespan and the energy consumption. Ander both deadline and budget constraints, Rizvi N et al. [18] formulated a Modified Fuzzy Adaptive Genetic Algorithm (MFGA) to minimize the makespan and improve resource utilization. A fuzzy logic controller had also been devised to control the crossover and mutation rates that prevented MFGA from getting stuck in a local optimum. Based on establishing a workflow task model Anbarkhan S H et al. [19] proposed an Enhanced PSO (EPSO) algorithm to meet the user’s time, cost, and CPU utilization requirements through the improvements to the initialization of particles and the design of adaptive fitness function of the particles. Tao S et al. [20] designed a novel scheduling algorithm DB-ACO for workflow tasks, which employed Ant Colony Optimization to optimize cost under budget and deadline. Chakravarthi K K et al. [21] presented a Cost-Effective Firefly-based Algorithm (CEFA) to minimize the makespan and execution cost of workflows simultaneously. For tackling workflow scheduling challenges in a cloud-edge environment, Bacanin N et al. [22] proposed an enhanced firefly algorithm, which optimized two objectives—cost and makespan. In recent years, meta-heuristic algorithms have shown great potential in workflow scheduling optimization, and more and more academic researchers are committed to using meta-heuristic algorithms to optimize scheduling decisions [23,24,25]. However, most meta-heuristic algorithms have the disadvantage of slow convergence speed in the early stages of iterative optimization, which affects search efficiency to some extent.

In order to further improve the efficiency of optimization algorithms for workflow scheduling, many studies have begun to explore hybrid optimization algorithms. Singh G et al. [26] presented a hybrid GA-modified PSO method to mapping tasks to VM, which minimized makespan, cost and energy consumption in heterogeneous cloud-fog computing environment. Shobeiri P et al. [27] developed a hybrid scheduling algorithm called PCP-ACO, which combined the Partial Critical Path (PCP) heuristic algorithm with the Ant Colony Optimization (PCP–ACO) algorithm to optimize the execution cost of a workflow under the deadline-constrained cloud environment. Mangalampalli S et al. [28] modeled the workflow scheduling problem with the combination of PSO and CS algorithms, achieving the effective task scheduling by considering task priority. Yin C et al. [29] introduced the NGACO algorithm based on the improvement of the crossover and mutation operators in GA and the combination of niche technology with the ACO algorithm, the optimization objectives consist of makespan, economic cost, total cost and load balancing. Combining the Seagull Optimization Algorithm (SOA) and the Grasshopper Optimization Algorithm (GOA), Mohammadzadeh A et al. [30] designed a hybrid multi-objective optimization algorithm denoted as HGSOA-GOA, which considered makespan, cost, energy, and throughput for scientific workflow scheduling. Pirozmand P et al. [31] proposed a hybrid scheduling algorithm called GSAGA, which had higher efficiency compared with the state-of-the-art by combining the general search capacities of the GA with the Gravitational Search Algorithm (GSA). Through the fusion of PSO, GA, and HEFT-based Initialization, Mikram H et al. [32] presented the hybrid HEFT-PSO-GA algorithm (HEPGA), which improved both makespan and Resource Utilization (RU) for scientific workflow scheduling in cloud computing environment. As mentioned above, hybrid methods have demonstrated good capabilities in workflow scheduling and injected new vitality into the research of workflow scheduling. The reasonable design of hybrid algorithms is crucial for improving optimization performance in multi-objective optimization of workflow scheduling.

In addition to heuristic, meta-heuristic, and hybrid optimization methods, in recent years, deep learning has also been applied to workflow scheduling in cloud computing. Sudhakar R V et al. [33] proposed an algorithm named Multi-Objective Reinforcement Learning-based Workflow Scheduling (MORL-WS), which can employ an agent and actor approach with action space and state space to optimize makespan and efficiency for Workflow Scheduling in dynamic cloud computing environment. Wang Z et al. [34] introduced a Deep Reinforcement Learning (DRL) framework for continuous workflow scheduling in heterogeneous cloud environments, which performs excellently in terms of average completion time and load balancing efficiency. Combining with Deep Reinforcement Learning (DRL) based on Proximal Policy Optimization (PPO), Chandrasiri S et al. [35] employed Graph Neural Networks (GNNs) to model the task dependencies within workflows, enabling the agent to adaptively and efficiently allocate cloud resources. Based on a Deep Reinforcement Learning algorithm called R-DQN, Pan J H et al. [36] proposed an online scheduling framework for multiple real-time workflows in dynamic environments, which aims to minimize workflow completion time while maximizing resource utilization. Luo L et al. [37] proposed an evolutionary strategy-guided Deep Reinforcement Learning (ES-DRL) scheduling model to solve the dynamic mixed flow shop scheduling problem. Ullah I et al. [38] presented multi-agent task allocation strategies and multi-agent reinforcement learning architectures, and examined their diverse applications and challenges in the Internet of Vehicles and the Internet of Things. Although these RL methods perform well in specific scenarios, they require extensive training data and incur high computational overhead. In contrast, our proposed HLWOA, as an unsupervised lightweight hybrid algorithm, is more suitable for conventional workflow scheduling scenarios.

## 3. Models and Problem Formulation

In this section, we will introduce the cloud resource model and the workflow model, followed by the formulation of the optimization problem.

### 3.1. The Cloud Resource Model

In the complex environment of cloud computing, cloud data center platforms are capable of providing a diverse range of virtual machine resources to meet the needs of different users. These virtual machines exhibit significant differences in rental prices and resource configurations, with key parameters such as CPU performance, memory capacity, and network bandwidth. Users can select appropriate virtual machines based on the specific requirements of their tasks and rent the required cloud resources from the cloud platform on a “pay-as-you-go” basis. This payment model allows users to pay only for the actual time they use, thereby effectively controlling cost.

To more specifically describe the cloud resource model, we assume the existence of a heterogeneous cloud resource pool comprising various types of virtual machines. Within this resource pool, there are W different types of virtual machines with varying processing capabilities available for selection, each equipped with a corresponding rental price. Typically, virtual machines with stronger processing capabilities come with higher rental prices. Moreover, each type of virtual machine can be utilized to execute workflow tasks, thereby offering users flexible scheduling options. For ease of description, we denote the set of available virtual machine types as $VM=\{VM_{1},VM_{2},VM_{3},\ldots ,VM_{M}\}$, where M represents the total number of leased virtual machines. Each virtual machine $VM_{k}$ has the following attribute characteristics:

$Price_{k}$: The rental price per unit time (e.g., per hour) for virtual machine $VM_{k}$.

$Speed_{k}$: The processing speed of virtual machine $VM_{k}$, typically measured by the size of tasks processed per second or computational capability.

$LeaseTime_{k}$: The rental duration of virtual machine $VM_{k}$, which refers to the length of time the user leases the virtual machine.

${Unit}_{k}$: The billing unit for virtual machine $VM_{k}$, such as hours or minutes.

$BW_{k}$: The network bandwidth of virtual machine $VM_{k}$, which indicates the speed of data transmission.

### 3.2. The Workflow Model

**Figure 1:**
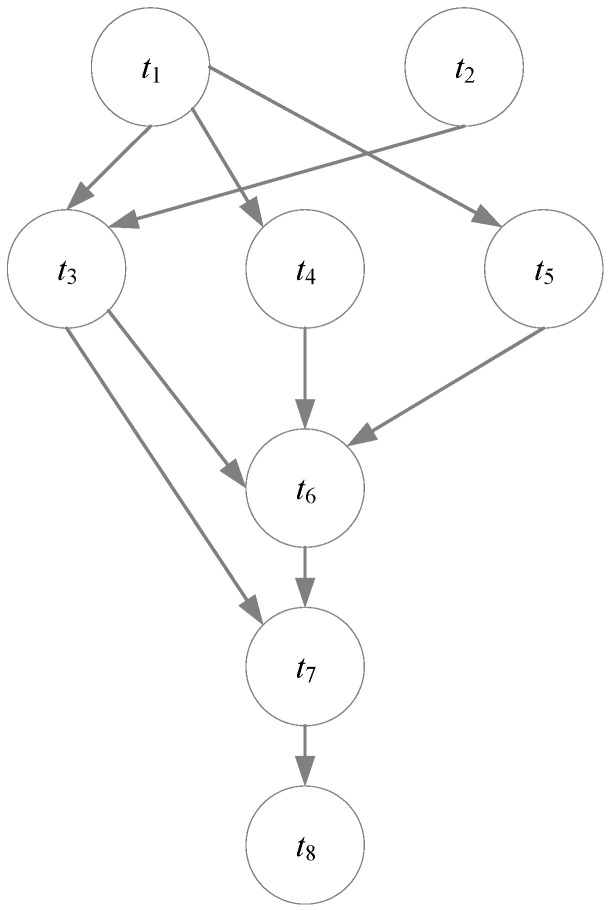
A workflow example of eight tasks.

The workflow model represents a workflow as a Directed Acyclic Graph (DAG), denoted as Dag=(T,E), where $T=\{t_{1},t_{2},t_{3},\ldots ,t_{N}\}$ is the task set and the edge set $E=\{e_{ij}|i,j=1,\ldots ,N\}$ represents the data dependencies between tasks. For any edge $e_{ij}\in E$, it indicates that task $t_{j}$ is the immediate successor of task $t_{i}$, and task $t_{i}$ is the immediate predecessor of task $t_{j}$. Each task $t_{i}$ can only start execution after all its immediate predecessor tasks have been completed and it has received all the data from the predecessor tasks. Additionally, each task $t_{i}$ has a task size $T\_size_{i}$, and each edge $e_{ij}$ has a weight $T\_D\_weight_{ij}$, representing the data transmission volume from task $t_{i}$ to task $t_{j}$. The workflow WF can be expressed as the following Equation (1). Figure 1 shows a DAG workflow consisting of eight interdependent tasks.

(1) $$WF=\{T,E,T\_size_{i},T\_D\_weight_{ij}\},\;\;\;i,j=1,\ldots ,N$$

In Figure 1, the task set *T* consists of 8 tasks (*t*_1_–*t*_8_), and the edge set *E* contains 10 directed edges. The arrow directions indicate the data flow and task dependencies. For example, the edge *E* (*t*_5_, *t*_6_) signifies that data are transmitted from Task 5 to Task 6; specifically, if *E* (*t*_5_, *t*_6_) = 9, then 9 units of output data from Task 5 are transferred to Task 6. Moreover, Task 6 cannot start until all its immediate predecessors (*t*_3_, *t*_4_, and *t*_5_) have completed execution and the required output data have been received.

### 3.3. Problem Formulation

In cloud computing platforms, an effective QoS-aware scheduler aims to find the optimal task-VM mapping scheme by optimizing scheduling decisions while satisfying users’ specific QoS requirements, thereby achieving a trade-off among multiple optimization objectives (e.g., workflow makespan and execution cost). This mapping relationship is formally defined in Equation (2).

(2) $$S=\{t_{i},VM_{k},Exe\_T_{k,i},Fin\_T_{k,i}|t_{i}\in T,VM_{k}\in VM\}$$

Here, $Exe\_T_{k,i}$ and $Fin\_T_{k,i}$ represent the execution time and finish time of task $t_{i}$ on virtual machine $VM_{k}$, respectively. To define the mapping relationship between tasks and virtual machines, this paper introduces $x_{i,k}$ as the scheduling decision variable, as specified in Equations (3) and (4). This binary variable indicates whether task $t_{i}$ is allocated to virtual machine $VM_{k}$: if assigned, $x_{i,k}=1$; otherwise, $x_{i,k}=0$.

(3) $$x_{i,k}=\left\{\begin{array}{l} 1,if\;task\;t_{i}\;\mathrm{is}\;\mathrm{on}\;VM_{k}\;\\ 0,\;\mathrm{otherwise} \end{array}\right.$$

(4) i=1,…,N,k=1,…,M

For any task $t_{i}$ in the workflow, let $T\_size_{i}$ denote the size of task $t_{i}$ and $Speed_{k}$ represent the processing speed of virtual machine $VM_{k}$. Then, the execution time $Exe\_T_{k,i}$ of task $t_{i}$ on virtual machine $VM_{k}$ is given by Equation (5):

(5) $$Exe\_T_{k,i}=\frac{T\_size_{i}}{Speed_{k}}$$

Additionally, let $Dtt_{i,j}$ denote the data transfer time from task $t_{i}$ to task $t_{j}$, where $TData_{i,j}$ represents the data volume transmitted between them, and Bw is the average network bandwidth of the virtual machine. The data transfer time $Dtt_{i,j}$ and the finish time of task $t_{i}$ on $VM_{k}$ are formally expressed by Equation (6) and Equation (7), respectively.

(6) $$Dtt_{i,j}=\left\{\begin{array}{l} 0,\;\mathrm{if}\;t_{i}\;\mathrm{and}\;t_{j}\;\mathrm{are}\;\mathrm{assigned}\;\mathrm{to}\;\mathrm{the}\;\mathrm{same}\;\mathrm{VM}\\ \frac{TData_{i,j}}{Bw},\;otherwise \end{array}\right.$$

(7) $$Fin_{-}T_{k,i}=Sta_{-}T_{k,i}+Exe_{-}T_{k,i}$$

Here, ${Sta\_T}_{k,i}$ denotes the start time of task $t_{i}$ on virtual machine $VM_{k}$, which implies that all direct predecessor tasks of $t_{i}$ have been completed. In Equation (8), $t_{l}$ represents a direct predecessor task of $t_{i}$, and $F_{l}$ indicates the completion time of $t_{l}$. Thus, ${Sta\_T}_{k,i}$ can be formally expressed by Equation (9). Furthermore, let $F_{i}$ represent the finish time of task $t_{i}$, which can be derived from Equation (10).

(8) $$pre(t_{i})=\{t_{l}|e_{li}\in E,t_{l}\in T\}$$

(9) $${Sta\_T}_{k,i}=\max\{F_{l}+Dtt_{l,i}|t_{l}\in pre(t_{i})\}$$

(10) $$F_{i}=\sum_{k=1}^{M}\sum_{r=1}^{N}({Sta\_T}_{k,i}+Exe\_T_{k,i})\cdot x_{i,k}$$

The makespan for workflow is defined as $F_{N}$, which represents the maximum value among all task completion times, and can be expressed by Equation (11):

(11) $$F_{N}=\max\{F_{i}|i=1,\ldots ,N\}$$

We then define the execution cost (Total_Cost) of a workflow as the sum of execution costs for all constituent tasks, formally expressed in Equation (12). The execution cost of each individual task is determined by the runtime duration on the allocated virtual machine and the unit price of the leased VM.

(12) $$Total\_Cost=\sum_{i=1}^{N}\sum_{k=1}^{M}x_{i,k}\cdot Price_{k}\cdot (Exe\_T_{k,i}+Dtt_{i,j})$$

Finally, the target problem we aim to solve is to design a workflow scheduling scheme that minimizes both the makespan and the execution cost of the workflow simultaneously. This multi-objective optimization problem for workflow scheduling can be formally modeled as Equation (13), subject to constraints Equations (14)–(16). Equations (15) and (16) ensure that every task in the workflow is allocated to one virtual machine during scheduling.

(13) $$\begin{array}{l} \min(Total\_Cost,F_{N})=\\ \min\left(\sum_{i=1}^{N}\sum_{k=1}^{M}x_{i,k}\cdot Price_{k}\cdot (Exe\_T_{k,i}+Dtt_{i,j}),\max\{F_{i}|i=1,\ldots ,N\}\right) \end{array}$$

(14) ∀k=1,…,M

(15) $$\sum_{i=1}^{N}x_{i,k}=1$$

(16) $$x_{i,k}\in \{0,1\}$$

## 4. A QoS-Aware Workflow Scheduling Optimization Approach

**Figure 2:**
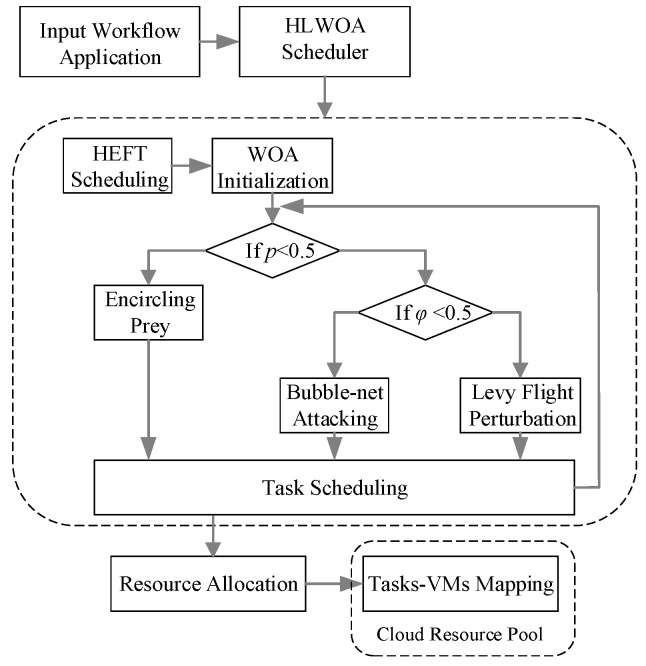
Workflow scheduling process by HLWOA in cloud environment.

In this section, we provide a detailed introduction to the hybrid HEFT-LWOA optimization algorithm for Workflow scheduling in cloud environments, which optimizes both makespan and execution cost simultaneously, as detailed in Section 4.1 and Section 4.2. Followed the time complexity analysis for the proposed method in Section 4.3. The scheduling process of the proposed method is illustrated in Figure 2, comprising two core components:

(1) HEFT Scheduling Based on Upward Rank Values: Under the premise of prioritizing the critical path, using the HEFT algorithm, the workflow tasks are sorted and allocated resources by calculating inverse topological order and performing dynamic dependency checks, thereby generating a scheduling scheme that minimizes completion time.

(2) LWOA Resource Allocation Optimization: A Levy Flight-enhanced WOA is utilized. The high-quality scheduling scheme generated by HEFT is used as an initial solution and incorporated into the initial population during the WOA initialization phase. Iterative searches are conducted based on the individuals of the initial population, whale individuals update their positions by choosing to encircle prey, perform bubble-net attacking, or undergo Lévy flight perturbations based on the thresholds set by the algorithm, and comparisons are made between the new current individual and the historically optimal individual to obtain the global optimal solution for resource allocation, thereby meeting the optimization objectives of minimizing makespan and cost.

### 4.1. HEFT Scheduling

HEFT is a workflow scheduling algorithm suitable for heterogeneous computing environments. Its objective is to minimize the overall completion time by reasonably arranging the execution order of tasks and allocating resource on multiple machines with different processing capabilities. Upward rank value is the core metric of the HEFT algorithm, which represents the longest time-consuming path length (including computation and communication overhead) from the current task to the exit task. The larger the value, the more the task is located on the critical path and therefore should be prioritized for scheduling. The calculation of the upward rank value follows the reverse topological order and is recursively represented by Equation (21). The upward rank value directly affects two key decisions of the scheduler: (1) Task priority sorting, where all tasks are sorted in descending order of the upward rank value to ensure that high ranked tasks are given priority in obtaining resources; (2) VM selection strategy, even if a VM currently has idle slots, the scheduler still chooses the one that allows the high-ranked task to finish earliest, not necessarily the VM that becomes available first. While this may increase the waiting time of some non-critical tasks, it markedly shortens the overall execution time along the critical path.

We employ the HEFT algorithm as a solution generation algorithm for a scheduling sequence within our proposed approach. The pseudocode of the HEFT scheduling algorithm is presented in Algorithm 1, the task scheduling process includes the following parts:

(1) Initialization phase

In the initialization phase of task scheduling, the upward rank value $rank_{u}(t_{i})$ of all tasks is initialized to 0. The priority of task $t_{i}$ is denoted by $rank_{u}(t_{i})$. During the initialization phase, for any task in the workflow, $rank_{u}(t_{i})=0$, preparing for the subsequent reverse topological sorting.

(2) Reverse topology sorting traversal

Traverse the tasks in the workflow in reverse topological order: Calculate the upward rank value for each task and ensure that the upward rank values $rank_{u}(t_{j})$ of all successor tasks $t_{j}$ of task $t_{i}$ have been computed when processing task $t_{i}$. The upward rank value of an exit task (a task with no successors) is simply its average computation time, as given by Equation (17).

(3) Average computation time

The average computation time is the average of the execution times of task $t_{i}$ across all machines, denoted as $\bar{w_{i}}$, which is used to eliminate the impact of virtual machine heterogeneity. It can be calculated using Equation (17). Here, W[i][k] represents the computation time of task $t_{i}$ on virtual machine $VM_{k}$, and m is the total number of virtual machines.

(17) $$\bar{w_{i}}=\frac{1}{m}\sum_{k=1}^{m}W[i][k]$$

(4) Communication overhead for successor tasks

Here, the communication overhead refers to the average data transmission time. $succ(t_{i})$ denotes the successor tasks of $t_{i}$. For each successor task $t_{j}$, $t_{j}\in succ(t_{i})$, the average data transmission time from $t_{i}$ to $t_{j}$, denoted as $\bar{c_{i,j}}$, which is calculated as shown in Equation (18). B[k][l] represents the bandwidth from virtual machine $VM_{k}$ to $VM_{l}$, and the calculation formula for the average network bandwidth $\bar{B}$ is given in Equation (19). Equation (20) records the maximum value of $rank_{u}(t_{j})+\bar{c_{i,j}}$ among all successor tasks, which is an important step in forming the critical path in task scheduling. “Critical path first” refers to prioritizing the scheduling of critical tasks that have the greatest impact on the overall completion time.

(18) $$\bar{c_{i,j}}=\frac{TData_{i,j}}{\bar{B}}$$

(19) $$\bar{B}=\frac{1}{m^{2}}\sum_{k=1}^{m}\sum_{k=l}^{m}B[k][l]$$

(20) $$\underset{t_{j}\in succ(t_{i})}{\max}(\bar{c_{i,j}}+rank_{u}(t_{j}))$$

(5) Update the upward ranking value

Starting from the exit tasks (tasks without successors), the upward rank values are updated recursively in reverse topological order. The final upward rank value of task $t_{i}$ is equal to its average task computation time plus the maximum successor overhead, which is specifically updated using the recursive Equation (21).

(21) $$rank_{u}(t_{i})=\bar{w_{i}}+\underset{t_{j}\in succ(t_{i})}{\max}(\bar{c_{i,j}}+rank_{u}(t_{j}))$$

(6) Generate task sequence

Sort tasks in descending order according to the upward sorting value and temporarily store them in a temporary list. Then, conduct dynamic dependency checking. Each time, select the first task in the temporary list whose all predecessors have been scheduled and add it to the final task list. Through this process, it is ensured that the tasks in the sequence always meet the dependency relationship.

(7) Assign VMs to the tasks

To optimize the overall makespan, tasks are allocated to the earliest available virtual machines according to their priority order. The algorithm iterates through all virtual machines $VM_{k}$, calculating both the start time and completion time of task $t_{i}$ on each VM. Then, the VM that enables the earliest completion of task $t_{i}$ is selected. The ready time of the chosen VM $VM_{k}$ is updated to reflect earliest completion time of $t_{i}$ on $VM_{k}$, ultimately yielding the resource allocation scheme.
**Algorithm 1** Workflow scheduling Based on HEFT Input: the workflow Dag=(T,E), W[i][k], B[k][l]
Output: A task_list sorted in descending order of Upward rank, the allocation of task to VM
       for each task $t_{i}$ in T do
               rank_u$[t_{i}$] ← 0
       end for
       for each task $t_{i}$ in reverse topological order of *Dag* do
               Obtain the average computation time $\bar{w_{i}}$ using Equation (17)
               if $succ(t_{i})$ is empty then
                      rank_u$\left[t_{i}\right]$$\;\leftarrow \bar{w_{i}}$
               else
                      Obtain the $\underset{t_{j}\in succ(t_{i})}{\max}(\bar{c_{i,j}}+rank_{u}(t_{j}))$ by Equations (18)–(20)
                      Update the $rank_{u}(t_{j})$ by Equation (21)
               end if
          end for
       task_list ← []
       temp_list ← sort T by $rank_{u}(t_{j})$ in descending order
       while temp_list is not empty do
               for each task $t_{i}$ in temp_list do 
                       all_pred_scheduled ← true
                       for each predecessor task $t_{k}$ of $t_{i}$ do
                               if $t_{k}$ not in task_list then
                                    all_pred_scheduled ← false
                                    break
                               end if
                       end for 
                       if all_pred_scheduled then
                               task_list.append$(t_{i}$)
                               temp_list.remove$(t_{i}$)
                               break 
                       end if
               end for
       end while
return task_list
       allocation ← {}
       machine_ready_time ← [0, 0, …, 0]
       for each task $t_{i}$ in task_list do
               earliest_finish_time ← ∞ 
               selected_machine ← −1 
               for each machine k in Machines do
                       Calculate ${Sta\_T}_{k,i}$ of $t_{i}$ on $VM_{k}$
                       Calculate $Fin\_T_{k,i}$ of $t_{i}$ on $VM_{k}$
                       Update the selected_machine based on earliest completion time
               end for
               Assign $t_{i}$ to the selected_machine $VM_{k}$
               Add this assignment to the allocation
               update the ready time of $VM_{k}$
       end for
       return allocation

### 4.2. Resource Allocation Optimization Based on Levy-WOA

In this section, the scheduling solution obtained by HEFT above is taken as an individual and incorporated into the initial population of WOA to accelerate the convergence speed of the WOA algorithm. The WOA with the introduction of the Levy Flight strategy is a hybrid optimization algorithm that combines the swarm intelligence search capability of the WOA and the global exploration characteristic of the Levy Flight, aiming to achieve more economical and efficient scheduling of the entire workflow under the optimization objectives of makespan and cost. The pseudocode of the algorithm is shown in Algorithm 2. The details are shown as follows.

(1) Population Initialization

During workflow task-to-VM scheduling, the algorithm first generates an initial population comprising population_size-1 whale individuals, where each individual represents a task-to-VM allocation scheme. These allocation schemes are created by randomly mapping tasks to VMs, ensuring diversity in the initial population to cover different regions of the solution space. Subsequently, the individual generated by HEFT is incorporated, resulting in a total of population_size initialized individuals, which lays the foundation for the subsequent iterative optimization.

(2) Fitness Evaluation

The optimization objective of this paper is to minimize the workflow completion time and execution cost. However, the values of makespan and cost may have different dimensions and ranges. If the two objective values are directly combined linearly, the value of one objective may dominate that of the other due to its dimension and range. This will cause the influence of some certain objective to be neglected in the optimization process.

To address this, in this paper, we use the makespan-equivalent total cost ME_TotalCost to replace the Total_Cost in (12), as expressed in Equation (22). Here, η is a user-defined time-cost equivalence factor, with a value between 0 and 1. Thus, we define the fitness function as Equation (23). This fitness function quantitatively evaluates scheduling solution quality, e.g., individuals with shorter makespan and lower cost yield higher fitness values and are thus prioritized as candidate solutions. Here, t denotes the iteration count. $\omega_{1}$ and $\omega_{2}$ represent the weighting coefficients for workflow makespan and execution cost, respectively, subject to the constraint $\omega_{1}+\omega_{2}=1$. By dynamically adjusting these weights, the algorithm controls the relative emphasis on each objective during optimization. For instance, increasing the weight of total cost prioritizes cost minimization, whereas assigning higher weight to makespan may reduce execution time at the expense of increased cost. This flexible weighting mechanism enables the algorithm to adapt to diverse QoS preferences by aligning with user-defined optimization priorities.

From Equation (23), the scheduling objective in this paper is transformed into maximizing the value of this fitness function, that is, the virtual machine allocation scheme corresponding to the individual with the highest fitness value is ultimately selected. The fitness value is calculated for each individual, and the fitness value of the current individual is compared with that of the historically optimal individual, and then the virtual machine allocation scheme corresponding to the higher fitness value is chosen as the current best solution. The calculation and evaluation of the fitness value provide a basis for decision making in the subsequent algorithm optimization, ensuring the search process progressively converges towards higher-quality feasible solutions.

(22) ME_TotalCost=η⋅Total_Cost

(23) $$Fitness(t)=\frac{1}{1+\omega_{1}\cdot F_{N}+\omega_{2}\cdot ME\_TotalCost}$$

(3) Encircling Prey

The ”encircling prey” phase refers to the process where whale individuals attempt to surround the current best solution (prey) during the search. In this stage, each whale updates its position based on the location of the current optimal solution, progressively contracting the search space to facilitate global exploration.

Let X(t) represent the position of the current whale individual, where $t\in [1,T_{\max}]$. In this paper, X(t) denotes the task-to-VM assignment at the *t*-th iteration. $X^{*}(t)$ is defined as the position of the current global optimal solution. A is a coefficient vector, calculated by Equation (25), which controls the direction and size of the encircling range. $\left|A\right|$ < 1 indicates local exploitation (moving closer to the optimal solution), while $\left|A\right|$ ≥ 1 indicates global exploration (moving away from the current area). *r* is a random number between 0 and 1. *D* represents the distance between the current individual and the optimal solution, as shown in Equation (26). *C* is a coefficient vector used to randomize the prey’s position and enhance exploration ability, as expressed in Equation (27). When an individual moves towards the direction of the current global optimal solution, the step size of the movement is controlled by the convergence factor *a*, which linearly decreases from 2 to 0 with the number of iterations, as calculated by Equation (24).

In the encircling prey phase, the WOA leverages the current best solution to guide the population towards high-quality regions. For workflow scheduling in this study, this mechanism dynamically adjusts task allocation schemes during the position-update process to identify the global optimum in the search space. The position update is formally defined by Equation (28).

(24) $$a=2-\frac{2t}{T_{\max}}$$

(25) A=2a⋅r−a

(26) $$D=\left|C\cdot X^{*}(t)-X(t)\right|$$

(27) C=2⋅r

(28) $$X(t+1)=X^{*}(t)-A\times D$$

(4) Bubble-Net Attacking

The bubble-net attacking phase simulates humpback whales’ spiral rising predation behavior, employing a logarithmic spiral path to locally refine the current solution. For workflow task scheduling, this phase enables fine-grained optimization by adjusting VM selections. For example, tasks are assigned to other virtual machines of similar type.

The bubble-net attacking phase can be implemented in two ways:

Contraction Encircling Mechanism: This behavior is achieved by reducing the value of a in Equation (25), which in turn changes the value of A. In other words, A is a random value within the interval [−*a*, *a*], where a undergoes a linearly decreasing iterative process from 2 to 0. When the random value of A lies within the interval [−1, 1], the individual will update its position towards the optimal position, and the update is realized through Equation (28).

Spiral Position Update: The individual spirally moves from its current position towards the position of the optimal individual, as expressed in Equation (29). Where $D^{\prime }$ represents the distance between the current individual and the optimal individual, b is the logarithmic spiral shape constant, usually set to 1, and *l* is a random number that determines the direction of the spiral, l∈[−1,1].

To simulate the synergistic mechanism of these two behaviors, a random number *p* is set, for example, with a threshold of 0.5. When *p* ≥ 0.5, the spiral update is performed; when *p* < 0.5, the contraction encircling mechanism is used to update the position. Through the synergy of spiral paths and contraction behavior, the bubble-net attacking phase achieves an in-depth exploration of potential areas. It is a key link in balancing the global exploration and local exploitation of the algorithm.

(29) $$X(t+1)=D^{\prime }\cdot e^{bl}\cdot \cos(2\pi l)+X^{*}(t)$$

(30) $$D^{\prime }=\left|X^{*}(t)-X(t)\right|$$

(5) Levy Flight Perturbation

Levy Flight perturbation is a random step-length generation mechanism based on the Levy Distribution. It enables the search for feasible solutions over a wide range in the solution space and introduces global exploration capability into the optimization algorithm. The Levy step length determines the probability of task reassignment. A large step length allows tasks to jump to a random VM, enhancing exploration ability and avoiding local optima. In this paper, the Levy step length Levy(β) is defined in Equation (31). α and β are defined as the scaling factor and the Levy index, respectively. The scaling factor α is usually set to 0.01, and the Levy index β is typically 1.5. *u* and *v* are random numbers from the standard normal distribution, defined in Equations (32) and (33), and Γ represents the standard Gamma function.

In this paper, to balance the abilities of exploration and exploitation, the probability φ is set (e.g., φ = 0.5, which is the threshold to trigger perturbation) to perturb individuals through the Levy Flight. The probability φ determines whether to trigger the perturbation. If the perturbation is triggered, the Levy step length is generated, and the task-to-VM mapping is updated, the position update is represented by Equation (34). Otherwise, the individual is updated according to the encircling or spiral mechanism of the WOA. Through φ and the Levy step length, the behaviors of the Levy Flight and the WOA optimization work together to update the position of the whale individual. This helps to avoid local optimal solutions and increases the search diversity and global exploration ability.

(31) $$Levy(\beta )=\frac{u}{{\left|v\right|}^{1/\beta }}$$

(32) $$u∼N(0,\sigma_{u}^{2}),\;v∼N(0,\sigma_{v}^{2})$$

(33) $$\sigma_{u}=\frac{\Gamma (1+\beta )\sin\left(\frac{\pi \beta }{2}\right)}{\Gamma \left[(1+\beta )/2\right]\beta \cdot 2^{(\beta -1)/2}},\;\sigma_{v}=1$$

(34) $$X(t+1)=X(t)+\alpha \cdot rand\cdot \left(\frac{u}{{\left|v\right|}^{1/\beta }}\right)$$

(6) Update the Individual and Output the Global Optimal Solution

After each iteration, the algorithm updates the current VM allocation scheme. Specifically, the start time of each task $t_{i}$ is recomputed based on the completion time of all its predecessor tasks and the data transfer times between dependent tasks. Then, for the newly generated scheme, calculate execution cost, workflow makespan and fitness values, and compare the fitness of the new scheme with the historically best solution to retain the scheme with higher fitness as the current optimum. Repeat this process until reaching the maximum iteration count, finally output the global best solution, namely the tasks-to-VMs mapping that simultaneously minimizes both makespan and cost.
**Algorithm 2** Levy-WOA workflow scheduling optimization algorithmInput: task_list, allocation obtained from HEFT, Dag = (T, E), VM_list, pre$(t_{i}),$
data_size$(t_{i},$$t_{j}),$ bandwidth$(VM_{k}$$,VM_{l}),$ population_size = 50, max_iterations $T_{\max}$ = 200, α = 0.01, β = 1.5, η = 1, $\omega_{1}$ = 0.5, $\omega_{2}$ = 0.5 

Output: the best solution best_schedule

population = []
for i = 1 to population_size-1 do
        schedule = {} 
        for task in task_list do 
                vm = random_select(VM_list)
                Obtain the start time ${Sta\_T}_{k,i}$ using Equation (7)
                   Obtain end time $Fin\_T_{k,i}$ of the current task and Equation (9) 
                schedule[task] = (vm, start_time, end_time)
        end for 
                population.append(schedule)
end for 
             population.append(allocation)
For each schedule in population do
      Calculate makespan $F_{N}$ of the workflow by Equation (11)
      Calculate total cost Total_Cost by Equation (12)
      Calculate fitness value using Equation (23) 
  end for 
  Return Initial “best_schedule” with the highest fitness value among the population
  Return Initial “best_fitness” with the hightest fitness value among the population 
                t = 0
while t$\;<\;T_{\max}$ do
                a←$a=2-\frac{2t}{T_{\max}}$p = rand(), φ = rand().
        for each schedule in population do 
                if p < 0.5 then 
                        Obtain a new schedule by Encircling Prey of WOA using Equations (24)–(28)
                else 
                     if φ < 0.5 then 
                           l = rand (−1, 1) 
                          for task $t_{i}$ in task_list: 
                                Obtain a new schedule by the spiral update in Bubble-net Attacking using Equations (29) and (30)
                     else 
                             for task $t_{i}$ in task_list: 
                                        Obtain a new schedule by Levy Flight Perturbation using Equations (31)–(34)
                       end if
               end if 
              Calculate” current_fitness” the fitness value of the current new schedule using Equation (23)
              If current_fitness > best_fitness then
              best_schedule ← the current new schedule 
              best_fitness ← the current_fitness 
              end if
end for
      t = t + 1
end while
Return best_schedule

When using WOA for workflow task scheduling, through distinctive step-length distribution, Lévy flight enhances global search capability. The role of the Lévy flight strategy in the proposed scheduling optimization algorithm can be summarized as follows.

(1) Heavy-tailed distribution: As shown in Equations (31)–(33), the Lévy step length Lévy(β) follow a heavy-tailed distribution. This endows the algorithm with a high probability of performing short-range fine-grained searches while retaining a small probability of long-range jumps. This property precisely matches the practical needs of cloud scheduling, where most tasks keep their current VM assignments and only a few critical tasks require cross-VM adjustments.
(2) Local Optimum Escape Mechanism: When the encircling or bubble-net attacking behavior of WOA causes the population to converge on a suboptimal solution (e.g., all individuals assign computationally intensive tasks to low-performance VMs), Lévy flight’s long step sizes can disrupt this uniformity. For instance, in a given iteration, a compute-heavy task may suddenly shift from its original low-performance VM to an entirely different high-performance VM. Even if the current best solution yields a makespan of 1200 s, Lévy perturbation could abruptly discover a new solution with a makespan of 1100 s while increasing the cost by only 2%.

### 4.3. Time Complexity Analysis

This section presents the time-complexity analysis of the proposed HLWOA, which consists of two phases: (1) the HEFT scheduling sequence generation stage and (2) the Lévy-WOA global optimization stage. During HEFT scheduling phase, the upward-rank computation, task sorting, and VM assignment are performed. The time complexity of HEFT is *O*(*N*^2^ × *M*), where *N* is the number of workflow tasks and *M* is the number of VM. In Lévy-WOA optimization phase, the time complexity of population initialization is *O*(*P* × *N* × *M*), with *P* being the population size. Each iteration selects the corresponding position-update method from encircling prey, bubble-net attacking and Lévy perturbation, and performs operations on all *P* individuals. Thus, the per-iteration complexity is *O*(*P* × *N*×*M*), the time complexity of the iteration optimization is *O* (*T*_max_ × *P* × *N* × *M*), *T*_max_ is the maximum number of iterations. Therefore, the overall time complexity of HLWOA is *O*(*N*^2^ × *M*)+ *O* (*T*_max_ × *P* × *N* × *M*).

## 5. Experimental Evaluation

### 5.1. Experiment Setup

(1) Experimental Setting:

To validate the effectiveness and efficiency of our proposed HLWOA, we conducted simulation experiments using WorkflowSim—an extension of the CloudSim cloud computing simulation platform. WorkflowSim is specifically designed for workflow scheduling emulation, which can simulate real cloud environments, support VM elastic configuration and on-demand billing models, and has a built-in DAG parser that can directly import workflows. We select three different types of heterogeneous virtual machines as the cloud resource model for this experiment, with an average network bandwidth of 10 MB/s. Detailed parameters of the cloud service VMs are provided in Table 1. 

**Figure 3:**
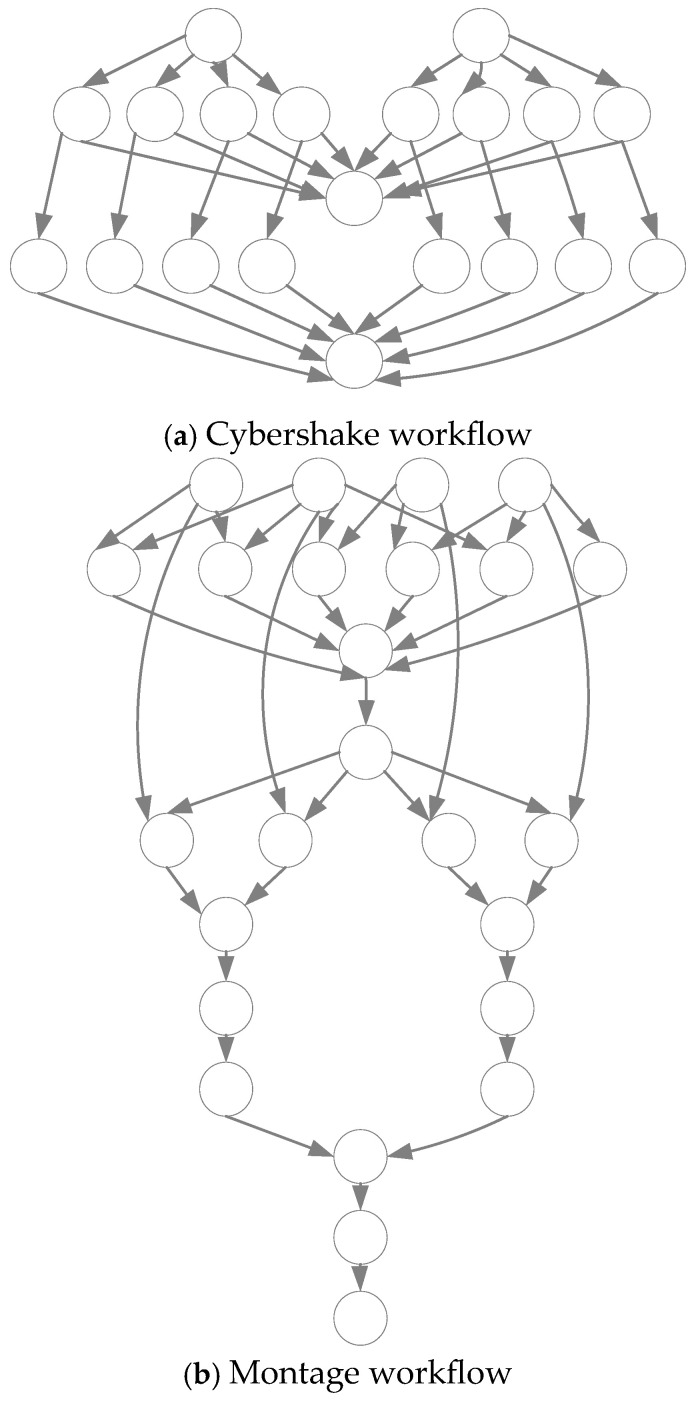
Workflow structure of Cybershake and Montage.

In this paper, we use two real-world scientific workflows [39] as the experimental datasets to verify the scheduling performance of the proposed algorithm HLWOA. These two workflows of different types are Cybershake and Montage, whose structures are shown in Figure 3. Comparison of workflows characteristics is presented in Table 2. The task scale of each workflow is set to 100 tasks and 1000 tasks. The parameter settings in the experiment are as follows: Through combinatorial testing of population size and iteration count parameters, it is determined that [50, 200] enables the HLWOA to achieve optimal convergence speed and solution quality—that is, a population size of 50 and a maximum iteration count of 200. Additionally, to ensure fairness in comparison, the comparison algorithms (HEPGA and WOA) and HLWOA all employ the same fitness function as the optimization objective evaluation function, as shown in Equation (23). Both $\omega_{1}$ and $\omega_{2}$ are set to 0.5 in this experiment, indicating equal emphasis on the optimization objectives of makespan and cost, with no explicit preference. The factor η is used to convert cost into a value equivalent to makespan, thereby balancing their importance in the optimization objective. Setting η = 1 signifies that the change in makespan is equivalent to the change in cost. Our experiments are conducted on a computer equipped with Intel (R) Core (TM) i7-8565U CPU@1.80 GHz, which runs on the 64-bit operating system of Windows 11.

(2) Comparison Algorithms

In this study, we selected several workflow scheduling algorithms as benchmarks for comparative analysis. The compared algorithms include

HEFT: Schedules tasks based on upward rank values to minimize the earliest finish time of all tasks.

HEPGA: A hybrid optimizer combining HEFT, PSO, and GA, as detailed in Reference [32].

WOA: Global exploration and local exploitation are realized by simulating the behaviors of whales encircling prey and bubble-net attacking to find the optimal solution.

These above workflow scheduling algorithms are compared with our proposed HLWOA to verify the advantages of the HLWOA.

(3) Performance Metrics

Makespan: Workflow completion time, as defined in Equation (11).

Cost: Total execution cost for a workflow, as defined in Equation (12).

Normalized Fitness: For the scheduling optimization problem addressed in this paper, in order to compare and visualize the quality of results in an overall manner, we employ the maximum normalization method to normalize the absolute fitness values. After applying normalization, the value with the maximum fitness is mapped to 1, with the remaining values falling between 0 and 1. Mathematically, maximum normalization is defined by the following formula: $\hat{x_{i}}=\frac{x_{i}}{\max(x_{j})},\;j=1,2,3,\ldots ,n$, where $\hat{x_{i}}$ represents the normalized value of $x_{i}$.

### 5.2. Experimental Results

**Figure 4:**
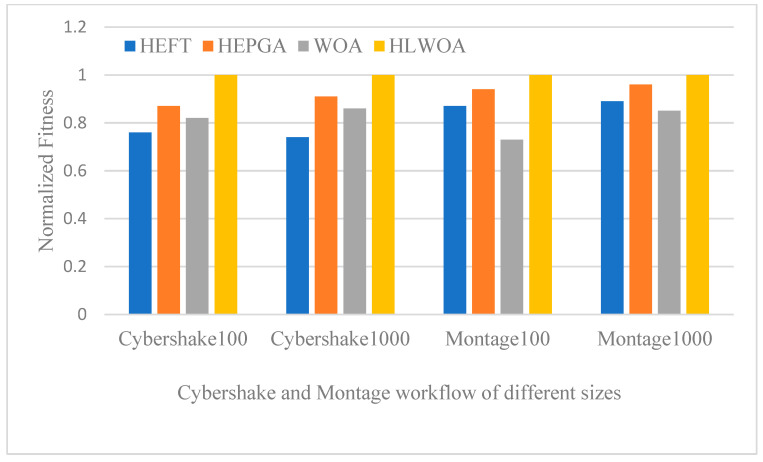
The normalized fitness comparison of different algorithms on various workflow.

In this section, we evaluate the performance of the HEFT, HEPGA, WOA, and the proposed HLWOAs using Cybershake and Montage workflows with different task scales. Table 3 presents the experimental results of the comparative algorithms on the Cybershake workflow, Table 4 shows the results on the Montage workflow, and Figure 4 illustrates the normalized fitness of the different comparative algorithms.

Table 3 shows the performance of different scheduling algorithms on the Cybershake workflow with different task scales. The scales for Cybershake workflow are 100 and 1000 tasks, respectively. Among all the compared algorithms, in terms of makespan, the makespan of HLWOA is the shortest under 100 tasks (1414.28), with improvements of 8.63, 6.07, and 2.74 compared to HEFT, HEPGA, and WOA, respectively. While under 1000-tasks HLWOA ranks third with a value of 11,330.01, and the makespan of HEPGA is the shortest (11,329.73). It can be seen that HLWOA is second only to HEPGA 0.28, with a difference of 0.002%, this small difference can be ignored in large-scale computing tasks and can be considered as a parallel optimum; But compared to HEFT, it still shortened by 5.31, and compared to WOA, it shortened by 3.67. Looking at cost, HLWOA demonstrates outstanding performance, achieving the lowest values under both 100 tasks and 1000 tasks, which are 4.63 and 24.81, respectively. Compared to HEPGA under 1000 tasks, this significant cost advantage is sufficient to compensate for the small gap in makespan. The comprehensive results of makespan and cost indicate that HLWOA has a significant advantage in cost optimization and also maintains strong competitiveness in execution time.

Table 4 shows the results of different scheduling algorithms applied to Montage workflows of different scales. Among all the optimization algorithms, as the number of tasks in the workflow increases from 100 to 1000, HLWOA achieves the lowest values in both makespan and cost, demonstrating outstanding performance. Specifically, the makespan value of HLWOA increases from 1204.05 to 11,215.69, and its cost value grows from 2.58 to 17.62, showcasing its excellent capability in handling large-scale Montage workflow tasks. In contrast, other algorithms such as HEFT, HEPGA, and WOA have higher makespan and cost values than those of HLWOA as the task scale expands. Based on the data in Table 4, HLWOA’s makespan and cost are both optimal for 100 tasks, reflecting fine scheduling ability of HLWOA for small-scale tasks. Under 1000 tasks makespan and cost of HLWOA continue to lead, indicating that it can still exhibit excellent steady-state optimal characteristics in large-scale Montage workflow tasks. These results highlight the effectiveness of HLWOA in balancing makespan and cost, which fully verifying the practical value and potential of HLWOA as a general cloud workflow scheduling algorithm among all the comparative algorithms.

As can be seen from Figure 4, among all the compared optimization algorithms, the HLWOA proposed in this paper has the highest fitness value on the Cybershake and Montage workflows with task scales of 100 and 1000. The normalized fitness value of HLWOA is 1, while the fitness values of other algorithms are all less than 1. It is evident that the proposed HLWOA outperforms the HEFT, HEPGA, and WOA algorithms in terms of the comprehensive performance of makespan and cost. We obtain the optimal resource allocation scheme using the HLWOA optimization algorithm. Even though the makespan value of HLWOA on the Cybershake 1000-task in Table 3 is not the smallest (higher than that of HEPGA), HLWOA has the lowest cost among all compared algorithms, and the gap in makespan between HLWOA and HEPGA has narrowed to a negligible level, proving that the global exploration ability of HLWOA is sufficient to continuously mine high-quality solutions in a large-scale search space.

## 6. Conclusions

This paper proposes a novel hybrid scheduling optimization algorithm for workflow scheduling in cloud computing environments, called HLWOA, which addresses the dual-objective optimization problem of simultaneously minimizing workflow makespan and execution cost.

Although numerous heuristic and meta-heuristic algorithms have been successfully applied to workflow scheduling in cloud computing (as reviewed in the related work), our proposed approach distinguishes itself through the following novel contributions: (1) we incorporate network bandwidth, the output data size of each task, the heterogeneous performance of virtual machines, and their rental prices to model a realistic scheduling environment, these features are mostly overlooked in existing studies. (2) We systematically derive a fitness function for the dual objectives of makespan and cost that introduces a time-cost equivalence factor, enabling dynamically adjusting objective weights to satisfy diverse user QoS preferences, this fitness-function design is novel. (3) The hybrid integration of HEFT, WOA, and Lévy flight leverages the strengths of each component, and as far as we know, this specific hybrid algorithm (HLWOA) has not been reported in the existing literatures.

The work of this article can be summarized briefly as follows. A solution generated by HEFT as an individual is incorporated into the initial population of WOA, and the Levy Flight strategy is introduced into the WOA algorithm, an efficient workflow scheduling was achieved by improving the quality of the initial solution and avoiding local optima during the optimization process. Comparative experiments are conducted on the Workflow simulation platform in cloud computing, using real-world scientific workflows of different task scales. The results demonstrate the effectiveness of the proposed algorithm in terms of makespan and cost.

In future work, we will primarily focus on the following areas of research: (1) developing adaptive virtual machine fault-tolerance mechanisms to address the issue of sudden VM failures in cloud data centers; (2) expanding the application scenarios to complex multi-cloud environments and designing effective optimization algorithms for multi-workflow scheduling; (3) studying workflow scheduling problems under different QoS constraints within real deployment cloud environments.

## Figures and Tables

**Table 1:** Configuration information of VMs in simulation experiments.

Type	vCPU	RAM (GB)	Bandwidth (Mbps)	$Speed_{k}$ (MB/s)	$Price_{k}$ ($/h)	Number
1	2	4	100	1.5	0.195	10
2	4	8	200	3.0	0.48	8
3	8	16	500	4.5	0.855	12

**Table 2:** Comparison of workflows characteristics.

Workflow	Application Area	Number of Tasks	DAG Structure	Data Dependency	Task Type
Cybershake	Seismic risk analysis	100/1000	Relatively large depth	High	Computation intensive
Montage	Astronomical image stitching	100/1000	Moderate depth	Moderate	I/O intensive

**Table 3:** The experiment results for Cybershake workflow.

Scheduling Algorithm	Makespan (Cybershake)		Cost (Cybershake)	
	100	1000	100	1000
HEFT	1422.91	11,335.32	4.75	25.51
HEPGA	1420.35	11,329.73	4.72	25.35
WOA	1417.02	11,333.68	4.71	24.96
HLWOA	1414.28	11,330.01	4.63	24.81

**Table 4:** The experiment results for Montage workflow.

Scheduling Algorithm	Makespan (Montage)		Cost (Montage)	
	100	1000	100	1000
HEFT	1205.01	11,219.02	2.65	17.81
HEPGA	1204.23	11,216.62	2.60	17.65
WOA	1206.02	11,217.58	2.62	17.79
HLWOA	1204.05	11,215.69	2.58	17.62

## Data Availability

Data is contained within the article.

## Abbreviations

All terms used in this manuscript.

**Abbreviations**	**Terms**
IaaS	Infrastructure as a Service
PaaS	Platform as a Service
SaaS	Software as a Service
VM	Virtual Machine
QoS	Quality of Service
HEFT	Heterogeneous Earliest Finish Time
DAG	Directed Acyclic Graph
WOA	Whale Optimization Algorithm
